# Effects of Lipid-Based Encapsulation on the Bioaccessibility and Bioavailability of Phenolic Compounds

**DOI:** 10.3390/molecules25235545

**Published:** 2020-11-26

**Authors:** Gulay Ozkan, Tina Kostka, Tuba Esatbeyoglu, Esra Capanoglu

**Affiliations:** 1 Department of Food Engineering, Faculty of Chemical and Metallurgical Engineering, Istanbul Technical University, Maslak, Istanbul 34469, Turkey; ozkangula@itu.edu.tr (G.O.); capanogl@itu.edu.tr (E.C.); 2 Institute of Food Science and Human Nutrition, Gottfried Wilhelm Leibniz University of Hannover, Am Kleinen Felde 30, 30167 Hannover, Germany; kostka@lw.uni-hannover.de

**Keywords:** polyphenols, curcumin, resveratrol, PMF, encapsulation, lipid-based delivery systems, bioaccessibility, bioavailability

## Abstract

Phenolic compounds (quercetin, rutin, cyanidin, tangeretin, hesperetin, curcumin, resveratrol, etc.) are known to have health-promoting effects and they are accepted as one of the main proposed nutraceutical group. However, their application is limited owing to the problems related with their stability and water solubility as well as their low bioaccessibility and bioavailability. These limitations can be overcome by encapsulating phenolic compounds by physical, physicochemical and chemical encapsulation techniques. This review focuses on the effects of encapsulation, especially lipid-based techniques (emulsion/nanoemulsion, solid lipid nanoparticles, liposomes/nanoliposomes, etc.), on the digestibility characteristics of phenolic compounds in terms of bioaccessibility and bioavailability.

## 1. Introduction

The dynamic market of so called “superfoods” grows steadily worldwide and offers new health-improving products regularly, although some of these foods have evolved into established products or food additives, e.g., Goji berries and Chia seeds [1]. While the demand for superfoods and healthier foods rose over the last years, the positive relationship between nutrition and health became more and more pronounced and forced the development of these kinds of products [2]. Moreover, the individual-related and specific nutrient supply, especially for the elderly, comes into focus. In 2050, nearly 16% of the world population will be aged over 65, whereby the demand for personalized functional foods will be increased in parallel as the population age [3].

Despite market growth in functional and healthier foods, their beneficial effects are controversially discussed, e.g., as reviewed by Marian (2017) [4]. In this review, nutrition studies with healthy humans consuming dietary supplements were summarized. Conclusively, most of the studies showed health-improving effects induced by the supplements, but at rather high doses which are unusual for the dietary intake [4]. For example, one of these food supplements is resveratrol, a naturally occurring phytoalexin that is synthesized in plants, e.g., grapes, as a response to injuries [5]. Resveratrol, as a food additive, possesses various health promoting effects including high antioxidant and anti-inflammatory potential, anticarcinogenicity in breast and liver tissue, prevention of osteoporosis, improving ischemic diseases and muscle regeneration, etc. [6,7]. Unfortunately, these health-improving effects have been mainly analyzed in cell culture studies or preclinical models, which makes the application of effective concentrations and substances more difficult in humans [6]. For example, the functionality of resveratrol is limited owing to its low bioavailability [5]. While the solubility of resveratrol in aqueous solutions is 3 mg/L, the solubility is enhanced to 50 g/L in ethanol, which results in a higher uptake and plasma concentration of resveratrol with a lipophilic-based food matrix. Besides, the bioavailability is too low to reach effective doses up to 1 g/day only by consumption of resveratrol-containing food. Theoretically, the consumption of about 3500 L of rose wine, 2600 kg of white grapes, up to 35,000 kg of peanuts or 2500 kg of apples per day were found to be necessary to reach these daily intake doses [6].

These results illustrate the need for developing new delivery systems for bioactive compounds, which show low bioavailability values [8], by altering the molecular structure or the physiochemical characteristics of bioactive compounds [7]. The pharmaceutical industry has developed technologies to improve drug delivery systems, which could be transferred to the food industry and may be also helpful for nanoscale delivery systems for food products [3]. The encapsulation of these compounds using nanoparticles, nanodelivery carriers or various emulsions could protect them against enzymatic degradation during digestion and increase the intestinal uptake, resulting in a higher gut concentration as well as increased plasma levels of encapsulated food additives [9].

The purpose of the present review is to ensure a critical assessment based on the effects of different lipid-based encapsulation techniques on the retention of phenolic compounds. In order to achieve this purpose, studies investigating the effect of encapsulation on the bioaccessibility and bioavailability of bioactive compounds were covered.

## 2. Overview of Phenolic Compounds Bioaccessibility/Bioavailability

**Figure 1:**
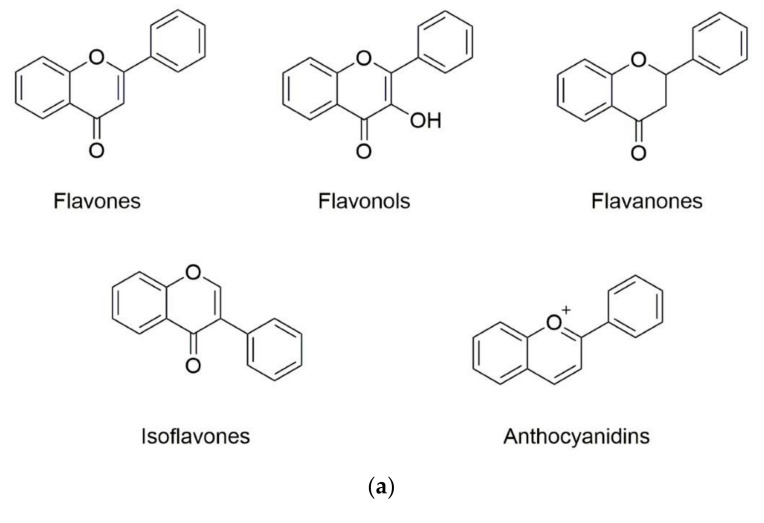

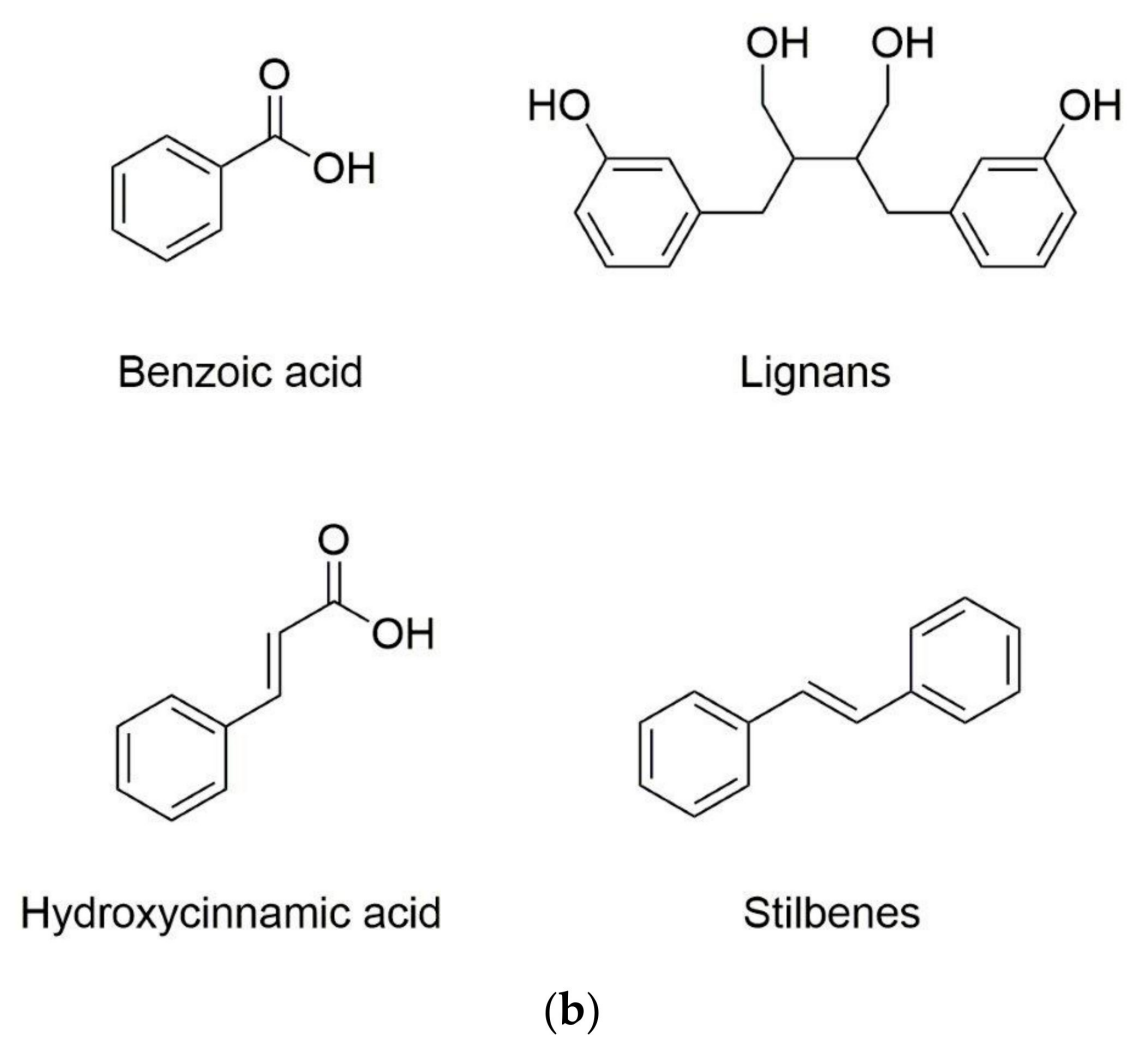
Basic structure of (**a**) common classes of flavonoids and (**b**) nonflavonoid-type phenolic compounds.

Phenolic substances are secondary metabolites which are present in a wide variety of foods such as fruits, vegetables, cereals, horticultural crops, legumes, chocolate, etc. and in beverages, i.e., tea and coffee [10]. Polyphenols with at least one aromatic ring and one or more hydroxyl groups can be categorized primarily as flavonoids and nonflavonoids. The basic structure of the common classes of flavonoids and nonflavonoids are shown in Figure 1. Flavonoids, as the most widespread and diverse group of polyphenols, can be further subdivided into flavonols (myricetin, quercetin, rutin, kaempferol etc.), flavones (aspigenin, luteolin, tangeretin etc.), flavanones (hesperetin, hesperidin, naringenin etc.), isoflavones (genistein, daidzein etc.) and anthocyanidins (cyanidin, delphinidin, malvidin, pelargonidin etc.) depending on the degree of hydroxylation, methoxylation, prenylation and glycosylation [11]. Nonflavonoids include diverse classes of polyphenols, such as stilbenes (resveratrol), lignans, hydrolyzable tannins and phenolic acids (hydroxybenzoic acids and hydroxycinnamic acids) [12].

Phenolic compounds have been used for the production of functional foods due to their many benefits to human health through antioxidant, anti-inflammatory, anticancer, antiobesity, antiviral, antibacterial, antiaging and/or antiallergenic activities [13]. In vitro studies reported that flavonoids showed a high anticancer potential by inhibition of the proliferation, metastasis and angiogenesis of tumor cell lines, while the process of apoptotic cell death was activated. Such beneficial effects were also detected in mice fed with citrus peel extract, rich in phenolic compounds. The skin and colon carcinogenesis as well as the tumor size and volume of mice suffering from prostate cancer was significantly reduced in treated animals. In addition to the health-improving effects of a phenolic-enriched extract, the specific effects of each individual phenolic compound can also be allocated; such as an anti-inflammatory potential of tangeretin and sinensetin or the suitability of hesperidin as an antioxidant [14].

Bioaccessibility and bioavailability of phenolic compounds are the main factors which effect the biofunctional properties and possible beneficial effects. Bioaccessibility as a clue for the release and solubility of bioactive compounds during gastrointestinal digestion for further uptake, is a considerable factor for bioavailability [15]. Furthermore, various external and internal factors are also determinants of the bioavailability of phenolic compounds. The external factors comprise the nature of the bioactive agent, including solubility, crystallinity, etc., as well as the composition and structure of the food matrix, while the internal factors include gender, age, health, nutrient status, and life phase [16].

The bioavailability of macronutrients such as carbohydrates, proteins, and fats are mostly higher than 90%. However, most of the phenolic substances, especially lipophilic ones, possess low levels of solubility, stability, bioavailability and target tissue specificity in the body [17] depending on their molecular and physicochemical characteristics [3]. Besides, each class of phenolic substances has different chemical structures, solubility (hydrophilic or lipophilic) and sensitivity to oxidation [13]. For example, the bioavailability of lipophilic bioactives such as curcumin, quercetin, rutin or polymethoxylated flavonoids (PMFs) is limited due to their poor solubility, high melting point and chemical instability [18,19,20,21]. Overall, it is essential to have high bioavailability leading to a sufficient substance concentration in the blood stream and finally enabling the production of effective functional foods with beneficial health effects [3].

Several approaches have been used to enhance the bioaccessibility and bioavailability of bioactive ingredients, including chemical modifications of the molecules, dosing formulations, combination with other dietary components as well as incorporating them within micro-/nanoparticle delivery systems [22]. The rapid dissolution of bioactive compounds within the gastrointestinal tract could be achieved by the relatively high surface area of these systems [23]. Consequently, there is a great attempt to develop phenolic compound loaded micro/nanoscale delivery systems by pharmaceutical and food industries. The important encapsulation techniques will be introduced in the following section.

## 3. Lipid-Based Delivery Systems

Encapsulation is a technology that has been utilized to protect active ingredients by a wall material to form capsules in nanometer to millimeter size [24,25]. Encapsulation promotes descended degradation from the external environment such as heat, light, moisture and oxygen during processing and storage. Further advantages are the prevention of interactions with other components, controlled release characteristics, the easier handling and masking of undesired sensory aspects as well as the increased bioavailability of the coated material [26,27,28,29].

A wide range of different food-grade carriers including polysaccharides, lipids, proteins and surfactants can be used for the fabrication of capsules by using different methods [28]. Numerous physical (spray drying, lyophilization, supercritical fluid precipitation and solvent evaporation), physicochemical (coacervation, liposomes and ionic gelation) and chemical (interfacial polymerization and molecular inclusion complexation) methods have been developed for the production of capsules. A schematic diagram of the different techniques is provided in Ozkan et al. (2019) [30]. The selection of the encapsulation technique is highly dependent on the thermosensitivity and solubility of the active compounds, type and hydrophilicity of the wall material to be used, interaction between core and wall materials as well as capsule specifications including physical, chemical and sensory qualities [30]. Furthermore, carriers need to be stable under stomach conditions and release the bioactive compound at the intestinal phase. Furthermore, encapsulation should contribute to the diffusion through the intestinal mucus layer so that bioavailability of bioactive ingredients encapsulated in the delivery systems can be greatly improved [31].

Among others, lipid-based delivery systems such as emulsion/nanoemulsion, solid lipid nanoparticles, nanostructured lipid carriers and liposomes are the techniques widely used to increase the solubility, stability, bioaccessibility and bioavailability of phenolic compounds [32,33]. Hereinafter, the lipid-based delivery systems will be explained in detail.

An emulsion system is a mixture of two immiscible liquids, one of which is being dispersed as small droplets into the other. When an aqueous solution is dispersed in oil phase, this is referred to as water-in-oil (W/O) emulsion, whereas an oil phase dispersed in aqueous solution is referred to as an oil-in-water (O/W) emulsion. Moreover, multiple emulsions such as oil-in-water-in-oil (o/w/o) or water-in-oil-in-water (w/o/w) have been developed [34]. Emulsions can either be used directly in the liquid state or be dried to form powders using spray drying or freeze drying [35,36]. They can be applied to encapsulate a high concentration of lipophilic active agents such as resveratrol, quercetin, curcumin and lipophilic vitamins [20,37,38,39]. Emulsion/nanoemulsion-based delivery systems have been successfully used in order to obtain controlled release and enhance the bioaccessibility/bioavailability of phenolic compounds [34].

Solid lipid nanoparticles and nanostructured lipid carriers are similar to the preparation of O/W emulsions, which have a fully or partially solidified lipid core [40]. The stability of the lipid nanoparticles depends on the number and type of lipids, the nature of the emulsifier(s), the initial droplet size and concentration, and the cooling conditions [34]. Solid lipid nanoparticles have some limitations such as low loading capacity and leakage during storage [41]. Nanostructured lipid carriers based on delivery systems have been recently developed to overcome these drawbacks [42]. Studies have suggested that the water solubility, bioaccessibility and bioavailability of lipophilic core materials as well as the stability against degradation can be improved by using lipid-based nanocarriers [33].

Liposomes and nanoliposomes are vesicles consisting of single or multiple bilayers composed of phospholipids which have one hydrophilic head and two hydrophobic fatty acid tails. Due to its biphasic character, liposomes are able to entrap hydrophilic, lipophilic, and amphiphilic molecules [41]. Different methods such as solvent evaporation, electroformation, thin film dehydration/rehydration, proliposome, membrane extrusion, dialysis, sonication, extrusion, freeze–thawing, microfluidization, high-pressure homogenization [43] as well as the superlip (supercritical assisted liposome formation) process can be applied to form liposomes [44].

## 4. Improving the Bioaccessibility of Phenolic Compounds by Means of Encapsulation

Bioaccessibility is the term used to define the amount of food compounds released from the food matrix in the gastrointestinal lumen, which is required for their intestinal absorption and bioavailability [45]. During digestion, food is exposed to three digestive phases including the oral, gastric and intestinal phase. In the oral phase, foods are submitted to mastication in a neutral environment with saliva, which contains amylase and mucin, to result in an oral bolus. The factors affecting mastication are food composition, food volume, number of chewing cycles, bite force, teeth condition, degree of hunger and habits [46,47]. In the gastric phase, the oral bolus is digested by gastric enzymes (e.g., pepsin and gastric lipase) and mechanical agitation (peristaltic movements) in acidic environmental conditions into a thick semifluid called chyme, which is further digested into macromolecules such as proteins, fats and polysaccharides prior to transfer to the small intestine [13]. Subsequently, in the intestinal phase, the digested food is further broken down into smaller constituents by bile salts and pancreatic enzymes (e.g., pancreatic lipase, trypsin, chymotrypsin) secreted from the intestinal mucosa at the environmental pH [48,49]. The nutrients can be absorbed in the small intestine, whereas the nonabsorbed digestion products pass to the large intestine for a fermentation process by the colonic microbiota. Finally, the remaining metabolites are excreted from the human body [50].

Bioaccessibility depends on various factors such as beverages consumed while eating, stomach contents, intestinal peristalsis, blood and lymph flow, physicochemical properties like pH, temperature and texture of the matrix and the basic structure of the phenolic compounds including the presence or absence of glycosylation, the type of conjugated sugar, the type of linkage to the aglycone, the site of glycosylation and the number of sugar moieties [51,52].

There are different methods used to examine the correlation between diet and health. However, in vitro models have been widely used to investigate the human digestive tract rather than in vivo (human or animal) models owing to ethical issues. In vitro digestion methods that are commonly used for food can be divided into static and dynamic methods. These systems are used to simulate the physiological conditions of the upper gastrointestinal tract (oral, gastric and small intestinal phases) [53]; thus provide some perception about the digestibility of controlled release systems and the bioavailability of functional compounds [54]. Static models, which use a constant ratio of food to enzymes and electrolytes and a constant pH for each digestive phase, have been widely used for food and pharmaceutical purposes due to their simplicity, practicality and low cost [54,55]. Static digestion methods have some shortcomings including a lack of the gradual addition of simulated gastric fluids and gastric emptying, constant enzyme activity regardless of the type of food as well as simulating the intestinal phase as one phase instead of the sequential duodenal, jejunal and ileal phases. Thus, the static in vitro digestion method should be used only to evaluate digestion endpoints rather than kinetic analysis of the different stages of the digestion process [53]. On the other hand, dynamic digestion models, comprising multichambered apparatus, are multistage systems to mimic, as close as possible, the human digestion conditions. Moreover, it is possible to follow the simulation of the physicochemical changes such as pH transitions, enzyme secretion alteration and peristaltic movements that occur during in vivo digestion [54,56]. Relatively complex structure, high expense to setup and maintenance are some of the limitations of this method [53].

The health benefits of phenolic compounds in foods vary with the level of their bioaccessibility. Besides, the protection of phenolics in the gastrointestinal tract could be improved by encapsulating them using carrier agents. Thus, up to date, bioaccessibility of a wide variety of food bioactives have been investigated. The impact of encapsulation techniques on the bioaccessibility of phenolic compounds are covered in Table 1.

With regard to increasing the water solubility of curcumin to improve its recovery, effective factors on the bioaccessibility of curcumin encapsulated within emulsion-based delivery systems have been analyzed by using the dynamic in vitro digestion model. Results highlighted that the bioaccessibility of curcumin depended on the type and amount of carrier lipids as well as the droplet size of the nanoemulsion-based delivery system, ranging from 1 to 58% [20].

To understand the effect of coencapsulation of (−)-epigallocatechin-3-gallate (EGCG) and quercetin in a W/O/W emulsion gels, gastrointestinal stability tests have been performed to analyze their bioaccessibility. According to the results, when simply suspended in water, the bioaccessibility of EGCG and quercetin were found to be 25.8 and 12.9%, respectively. In contrast, when coencapsulated in W/O/W emulsion gels, bioaccessibility of both EGCG and quercetin increased to around 48.4 and 49%, respectively [57].

Aditya et al. [33] investigated the influence of the physical state and composition of the lipids on the achievement of quercetin-loaded lipid nanocarriers by means of solid-lipid nanoparticles, nanostructured lipid carriers and lipid nanoemulsions, respectively. The results of this study provided a promising perspective for the use of nanostructured lipid carriers and lipid nanoemulsions with the highest bioaccessibility values (~60%) compared to solid lipid nanoparticles (~35%) and free quercetin solution (~7%). Similar trends were also ensured by Pool et al. [38]. Recovery of quercetin was determined by a dynamic in vitro gastrointestinal model. Results highlighted an enhancement in the quercetin bioaccessibility from <5% in bulk water to 53% with 0.1 mg·mL^−1^ and 29% with 0.5 mg·mL^−1^ loading capacity when it was incorporated in the nanoemulsion. Ni et al. [58] provided a 33.6% bioaccessibility value of quercetin in a nanostructured lipid carrier, which is higher than that obtained in the bulk water (<2%). Moreover, eudragit, an anionic copolymer based on methacrylic acid and ethyl acrylate nanoparticles were also utilized to increase the stability and solubility of quercetin in the gastrointestinal tract. Pool et al. [59] studied the formation of a delivery system for encapsulation of quercetin by using a solvent displacement method. After simulated gastrointestinal digestion, quercetin release was around 7% for free quercetin dispersed in water, and around 22% for quercetin encapsulated within polymeric nanoparticles, indicating less increase in the recovery of quercetin than among other encapsulation techniques. In another study, Sessa et al. [37] obtained chemically stable (no changes in the quantity and quality) resveratrol-loaded nanoemulsions during the gastric and intestinal digestions.

PMFs are one type of flavone compound which possess methoxy groups on the flavonoid backbone and exist almost exclusively in the peel of numerous citrus fruits [65]. PMFs exhibit a wide spectrum of biological activity, including anti-inflammatory, anticarcinogenic, antibacterial, antioxidant and neuroprotective effects [66,67]; metabolic modulations [68]; protection against cardiovascular diseases [69,70]; reducing serum triacylglycerol, very low-density lipoprotein (VLDL), and low-density lipoprotein (LDL) levels [70]. Although PMFs have various health benefits, the potential applications of these compounds are limited due to their high hydrophobicity, low water solubility, high melting point and crystalline structure [21,71]. Due to the fact that the oral efficacy of compounds is tightly dependent on aqueous solubility, gut wall permeation, and metabolic stability, one of these strategies could be implemented to improve bioaccessibility and bioavailability [72].

Tangeretin belongs to the class of PMFs [73]. Chen et al. [60] investigated the effect of dietary lipids on the gastrointestinal fate of tangeretin-loaded zein nanoparticles. The recovery of tangeretin was found to be related to the concentration of the co-ingested lipid phase. The bioaccessibility of the delivery system was enhanced from 15 to 37% with the use of a 4% initial oil concentration. In another study, different in vitro models were used to evaluate the effect of emulsification on the bioaccessibility of tangeretin. In vitro lipolysis showed that bioaccessibility of emulsified tangeretin increased from 9.7 to 29.3% when compared with unprocessed tangeretin oil suspension. Besides, according to the dynamic in vitro gastrointestinal model (TIM-1) results, the bioaccessibility of tangeretin increased 2.6-fold when it was incorporated into the viscoelastic system rather than in the oil suspension [61]. Similarly, a recent study [62] compared the effects of high internal phase emulsions (HIPE) stabilized by whey protein isolate—low methoxy pectin complexes and medium chain triglyceride (MCT) oil as a suspension on the bioaccessibility of tangeretin using in vitro lipolysis and dynamic in vitro intestinal digestion studies. The in vitro lipolysis results revealed that the bioaccessibility of tangeretin in HIPE-complexes was increased 2-fold compared to that of the bulk oil. Additionally, the gastrointestinal model TIM-1 indicated a 5-fold increase in the total bioaccessibility of tangeretin compared to PMFs in bulk oil.

Nobiletin, another kind of PMF, was also studied to improve its bioaccessibility. Ning et al. [63] fabricated 5-demethylnobiletin (5-DN) loaded selenium-enriched peanut protein nanoparticle-stabilized Pickering emulsion. The bioaccessibility of 5-DN was found to be higher (18.3%) with emulsion than in bulk oil (9.2%). Similar results were found by Wijaya et al. [62]. 1.5- and a 2-fold increase in the bioaccessibility of nobiletin was obtained within HIPE-complexes compared to within bulk oil after in vitro lipolysis and the gastrointestinal model TIM-1 digestion, respectively. Furthermore, with regard to increasing oral bioaccessibility of PMFs from aged citrus peel extracts, lipid-based delivery systems have been developed [64]. Compared to the samples in bulk oil, the bioaccessibilities of PMFs in the nanoemulsion and Pickering emulsion were enhanced 14-fold with the use of a lipolysis model. On the other hand, results from the TIM-1 system demonstrated a 2- and 4-time increase in the bioaccessibilities of PMFs in the nanoemulsion and Pickering emulsion rather than that of bulk oil, respectively.

In conclusion, to generate a delivery system to enhance the solubility, stability, bioaccessibility and controlled release of a phenolic compound, factors including the solubility and thermal sensitivity of the phenolic compound, ratio and interaction between wall and core material as well as the phenolic loading ratio should be considered.

## 5. Intestinal Transport Mechanisms and Effective Factors on Phenolic Compound Bioavailability

**Figure 2:**
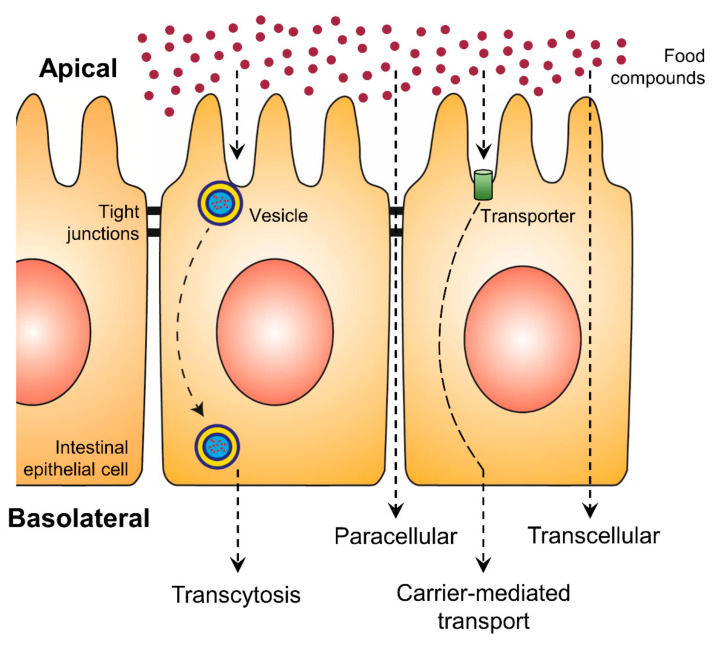
Uptake of food compounds by intestinal epithelial transport mechanisms from the gut lumen (apical side) to the blood vessel (basolateral side).

An increase in bioaccessibility by encapsulation is the initial step for higher exploitation of phenolic compounds. Nevertheless, the bioavailability is equally essential and represents the second step, which can be positively affected by encapsulation. With increasing intestinal absorption of phenolic compounds, their biological activities will be increased. The intestinal epithelial transport mechanisms can be divided into four different routes: the paracellular route, the transcellular route, the carrier-mediated transport and transcytosis (Figure 2) [74,75]. While on the transcellular route substances diffuse through the membranes and the intracellular space of the epithelial cells, on the paracellular route ions and small molecules can passively diffuse through the tight junctions. More complex and hydrophilic molecules use vesicles along transcytosis or they bind to specific transporters, which are integrated into the membrane of the intestine, in the case of carrier-mediated transport [75]. Phenolic compounds are mainly absorbed by passive diffusion, where the lipophilicity and molecular weight of each molecule are crucial [12]. While such substance-specific features represent the first group of effective factors on polyphenolic bioavailability, the second group consists of all possibly consumed compounds of the dietary matrix, which may influence the digestion processes and the composition of the person-related microbiome.

One of the most important factors for high bioavailability is the degree of polymerization as well as the methylation of the phenolic compound [76,77,78,79]. (−)-Epicatechin, a flavan-3-ol, possesses moderate bioavailability in in vivo studies with an average absorption of 23% after 90 min [76] or 46% after 2.5 h [80]. While 95.8% of transferred flavanol-related compounds were identified as (−)-epicatechin, the epicatechin dimers B2 and B5 showed a significantly lower content of <1% of the total transferred value [76]. Similar results were detected for further flavan-3-ols, whose monomers can directly be absorbed in the small intestine. More complex substances, e.g., polymeric forms will be transferred to the colon, where gut bacteria metabolize the compounds by glucuronidation or sulfation prior to absorption [79]. Unfortunately, the health-improving potential of these microbial-derived metabolites are largely unknown. Whether a flavone will be directly absorbed or possibly metabolized, depends on the methylation state likewise. Wen and Walle [77] and Wen and Walle [81] analyzed the stability of methylated and nonmethylated flavones in addition to liver S9 fraction or in the presence of human hepatocytes. The methylated compounds showed a high resistance against metabolization in all assays compared to the nonmethylated forms, suggesting that the methylation of flavonoids eventually protects them from metabolization and excretion [81]. In further in vitro transport experiments, up to 8 times higher absorption rates were documented for methylated compounds, while the rate of the nonmethylated forms was lower and correlated with their high potential of metabolic transformation [77]. Therefore, the replacement of hydroxyl groups by methylated groups may be another suitable method for increasing phenolic compound bioavailability.

The metabolization of phenolic compounds by the microbiome and/or intestine epithelial cells plays an important role in bioavailability. Nevertheless, elements of the dietary matrix can influence the bacterial growth and the composition of the microbiome, resulting in different digestion and metabolization pathways. Roowi et al. [82] detected a high content of phenolic acids (3‑hydroxyphenylacetic acid, 3-hydroxyphenylhydracrylic acid, dihydroferulic acid, 3-methoxy-4-hydroxyphenylhydracrylic acid and 3-hydroxyhippuric acid) in the urine of participants after consumption of orange juice, which corresponded to 37% of total ingested flavanones. The excretion of these acids was significantly reduced by parallel consumption of orange juice with yoghurt, suggesting an increased metabolization by gut bacteria [82]. Similar to the effects of yoghurt, the naturally occurring dietary fiber pectin influenced the metabolic activity and/or composition of the intestinal flora and induced a higher quercetin plasma concentration after rutin digestion [83]. Moreover, glucose and insulin are effective factors on bioavailability. While the total anthocyanin content in red wine and red grape juice was comparable, the uptake of anthocyanins of red grape juice was significantly higher than that of red wine, which might be due to the lower glucose content in red wine [84]. Such synergistic effects of glucose and the phenolic compound absorption may be based on the stimulation of bacterial growth, whereby the bacteria use glucose as an energy source [83] or alternatively the high glucose content induces the release of insulin, which is able to influence the microbiome and the phenolic bioavailability [85]. Further, bacteria-independent effects may be induced by protein complexes and fat-enriched diets. Proteins, e.g., the salivary protein histatine 5 can bind phenolic compounds and form insoluble complexes, which are related to reduced absorption [86]. Otherwise, experiments with milk protein had no effect on the uptake of cocoa polyphenols [87]. However, a high dietary fat content is associated with greater absorption in a dose-dependent manner [88]. Lesser et al. [89] analyzed the bioavailability of quercetin in pigs, whereby the dietary fat content was increased from 3 to 17%, resulting in an enhanced absorption of 50%. It is assumed that quercetin was incorporated in micelles, derived from the dietary fat, followed by absorption in the small intestine due to a higher solubility [89]. This principle of using a lipid carrier is already used as an effective encapsulation method for higher phenolic absorption.

## 6. Improving the Bioavailability of Phenolic Compounds by Means of Encapsulation

In order to compare the absorption efficiency of encapsulated vs. nonencapsulated compounds, several in vitro and/or in vivo assays were performed, followed by substance-specific quantification, e.g., LC-MS/MS [90,91,92]. For the in vitro assays, the absorption and/or the transport through an epithelial membrane were analyzed, using intestinal epithelial cells like the human colon adenocarcinoma cell line Caco-2 [93,94]. In the case of the absorption study, e.g., described by Jain et al. (2013) [95], Caco-2 cells were cultured and treated with encapsulated phenolic compounds. Afterwards the cells were washed to remove nonabsorbed material, followed by cell lysis and a substance-specific quantification of the intracellular content. Finally, the intracellular concentration was compared to the results of the nonencapsulated compound as well as the treatment concentration. Caco-2 absorption studies are fast and easy methods for analyzing bioavailability. Nevertheless, the experimental design contains undifferentiated cells, without brush border formation, which rather mimic mature enterocytes in the human physiology [96]. Moreover, for the health-improving effects induced by phenolic compounds, the transport of these compounds through the intestine to the blood flow is essential. Therefore, instead of uptake, the transport rate would give more insight into the bioavailability and efficiency of encapsulated substances.

**Figure 3:**
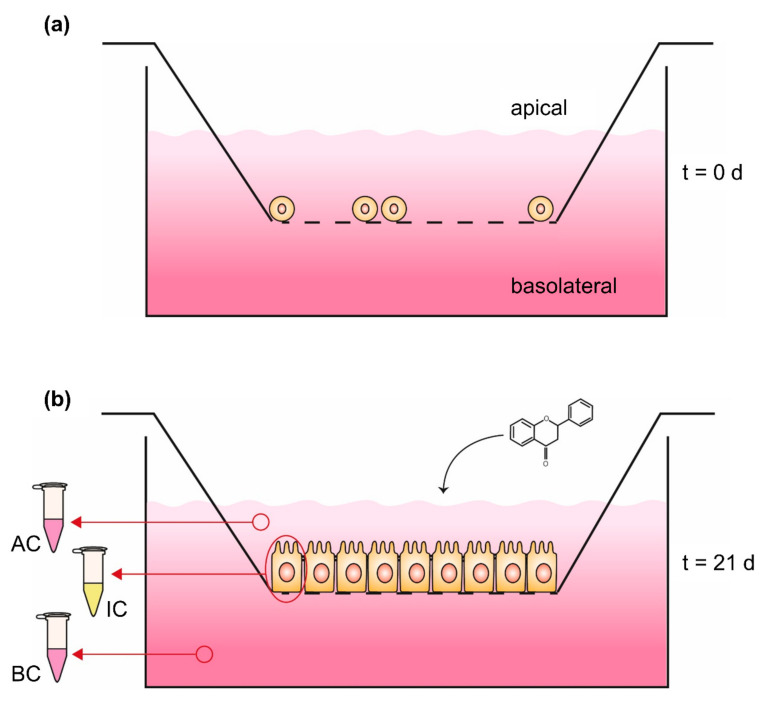
Caco-2 monolayer transport system (**a**) at seeding time (t = 0 days) and (**b**) 21 days (t = 21 days) after seeding. Phenolic compounds will be applied in the apical compartment followed by quantification of the content in both compartments as well as the intracellular concentration; AC: medium sample of apical compartment, IC: washed and harvested cells for quantifying intracellular content, BC: medium sample of basolateral compartment.

The Caco-2 monolayer transport system has been established to investigate bioavailability with a much more complex in vitro model (Figure 3). Thereby, an insert, which represents a downsized version of a cell culture dish, is hung, e.g., in a 6-well of a plate, resulting in the separation of the well into an upper compartment (volume of the insert) and a lower compartment (volume of the 6-well). The ground of the insert consists of a 10 µm thick membrane, made out of polyester or polycarbonate with µm-sized pores, which enable an exchange of molecules and media components but not cells between both compartments [94]. On the membrane of the upper compartment, intestinal cells like Caco-2 can be cultured and differentiated to receive an intestinal epithelium, consisting of an enterocyte monolayer with tight junctions and brush border formation [96]. The differentiation of Caco-2 cells occurs spontaneously by reaching 100% of confluence [97] and is completed after 16–21 days of further cultivation, resulting in an intestinal membrane similar to the epithelium of the small intestine [93,98]. In this Caco-2 monolayer transport system, the upper compartment is comparable to the intestinal lumen or the apical side of the gut membrane, while the lower compartment is comparable to the blood vessels or basolateral side of the gut membrane [93]. Thus, the in vivo processes can be simulated in more detail and in addition to the absorbed phenolic content in the cells, the concentration in the apical and basolateral compartment can be quantified [94]. Yee [99] verified the suitability of the Caco-2 monolayer transport system with a high correlation between the absorption results in humans and the permeability coefficient of the in vitro model. In spite of the good applicability of the Caco-2 monolayer transport system, this in vitro model can be extended with methotrexate-induced differentiated HT-29 cells, originating from a human colon adenocarcinoma, to get a mucus-secreting coculture [94,100,101]. The cultivation of colon epithelial cells in coculture with HT-29 goblet cells and mucus formation is a more sophisticated model for bioavailability, especially since the mucus represents a second physiological diffusion barrier, influencing the absorption time of digested compounds [94,101]. An overview of in vitro studies analyzing the uptake of encapsulated phenolic compounds in Caco-2 absorption studies or the Caco-2 monolayer transport system are shown in Table 2.

The Caco-2 monolayer transport system is a useful tool for intestinal transport studies, nevertheless, factors like the flow rate or gastrointestinal transit are not considered. For analyzing the digestion and uptake of food compounds, in vivo studies combine the influence of encapsulation on bioaccessibility and bioavailability, whereby for each step and organ the phenolic concentration can be quantified, e.g., as done by Augustin et al. [105]. Studies related to nutrition and health were mainly performed using humans, mice or rats as model organisms [106], which run through several periods of consumption and/or fasting. The human nutrition studies of Vitaglione et al. [91] and Mueller et al. [92] started with a wash-out period over days, consisting of a phenol-free diet, followed by fasting for several hours [91], and the consumption of the encapsulated/nonencapsulated phenolic compounds. Nallamuthu et al. [107] studied the uptake of chlorogenic acid in rats after fasting for 14–15 h. Ideally, all participants or test animals should run through all kinds of encapsulated samples, separated by a further wash-out period with normal consumption habits [108], to directly compare and evaluate the effects of encapsulation methods. In order to quantify such increased or decreased effects on the phenolic absorption, blood, urine and/or fecal samples are collected regularly [107], which enable a time-dependent distribution and excretion analysis of the test substance within the digestion system and the blood flow. For a whole-body distribution analysis including the separation of stomach, small intestine, cecum, colon and liver, Augustin et al. [105] fed rats with radiolabelled phenolic compounds and measured the radioactivity in each organ 3, 6, 12 and 24 h after dosing. While in this study the rats needed to be dissected, Penalva et al. [106] used a specific gamma camera to visualize the radiolabelled nanoparticles in the gastrointestinal tract of test animals similar to magnetic resonance imaging. Independent of the imaging method, the transport of radiolabelled, encapsulated compounds could be detected in more accuracy, especially if the encapsulation led to a slower but sustained absorption as described by Nallamuthu et al. [107] and Liu et al. [109]. Detailed results of the above-mentioned studies as well as further bioavailability experiments for the encapsulated phenolic compounds are shown in Table 3. 

Curcumin becomes more and more popular as a food additive and nutritional supplement due to its antioxidant and anti-inflammatory effects [111]. Nevertheless, it is weakly soluble in water, which restricts its bioavailability as well as its health-improving potential after consumption [112]. Lu et al. [64] significantly increased the uptake of curcumin by encapsulation using milled starch particles in the form of a Pickering emulsion. Compared to a standard curcumin solution dissolved in DMSO, the emulsion led to a higher intake in the Caco-2 absorption study [64]. While the results of in vitro models are limited and cannot be directly transferred to the digestion system of animals and humans [95], further studies reported various encapsulation techniques which were tested in vivo, followed by the quantification of their bioavailability. Curcumin was encapsulated with cellulose derivatives in oil [108], organogel [110], sophorolipid micelles [90] or a specific protease inhibitor from soybeans [109]. In all these experiments, the bioavailability of curcumin was analyzed by measuring the serum concentration over several hours after consumption, in comparison to the nonencapsulated polyphenol- or curcumin-loaded sodium caseinate nanoparticles [109]. After encapsulation, the uptake of curcumin was increased in the range of 3- to 9-fold higher serum concentrations, in detail, the cellulose-oil mixture (7-fold higher) and the organogel technique (9-fold higher) were found to be the most effective methods for encapsulation [90,108,109,110]. Although the experiments were partly done with mice and rats, similar uptake-improving effects were assumed for human bioavailability of encapsulated curcumin.

As mentioned above, in vivo studies combine the results of bioaccessibility and bioavailability. Therefore, the described higher serum concentrations of encapsulated curcumin also give an insight into a constant or possibly higher bioaccessibility induced by nanoemulsions, nanoparticles or organogels. Such a combination of higher occurrence and uptake of a curcumin-loaded sophorolipid-coated nanoparticle was measured by Peng et al. [90], who detected 2.7-fold higher bioaccessibility and 3.6-fold higher serum concentration than free curcumin. In this case, the higher bioavailability was mainly affected by the increased stability and occurrence of the curcumin due to the encapsulation and less affected by uptake. In contrast, an O/W emulsion of resveratrol showed a 2- to 4-fold increased uptake, resulting in significantly higher concentrations in the blood and liver of rats, while no effects on the bioaccessibility of encapsulated resveratrol were detected [105].

Another important factor of bioavailability is the particle size as well as the use of phospholipids for encapsulation. Sessa et al. [104] analyzed the in vitro uptake and permeability of multiple resveratrol emulsions, which were all based on peanut oil but differed in their composition of soy lecithin and droplet size. An increase in mean droplet size was negatively associated with the permeability but positively associated with cellular uptake. While smaller particles (128 or 137 nm in mean) were transported through the Caco-2 monolayer and accumulated in the basolateral compartment, it was assumed that larger particles (211 or 235 nm in mean) remained in the cells and could be responsible for the higher uptake contents. For permeability, the use of phospholipids in the encapsulation are beneficial compounds, resulting in a better interaction between the nanoemulsion-based delivery system and the cell membrane. By generating a phospholipid layer from soy lecithin, the permeability of encapsulated resveratrol was significantly higher compared to particles consisting of Tween 20 and glycerol monooleate. Moreover, in the same study, the degradation of resveratrol in water could be reduced to 15–25% by encapsulation with soy lecithin, while the nonencapsulated substance showed a degradation rate of 52%. To sum up, small particles in combination with phospholipids enables the inhibition of degradation processes, as well as a high permeability of substances with low water solubility like resveratrol, curcumin and anthocyanins [104].

## 7. Potential Risk of a Higher Phenolic Bioavailability

Although phenolic compounds are widely used for disease prevention due to their antioxidant properties, there is some evidence for toxic potential in polyphenols and flavonoids as reviewed by Kyselova 2011 [113]. For instance, flavonoids showed a mutual influence with cytochrome P450 monooxygenases (CYPs), which are essential enzymes in metabolism and in the activation of ingested food compounds, medications or environmental toxins like polycyclic aromatic hydrocarbons (PAHs) [114]. Flavonoids, e.g., quercetin and diosmin are able to increase the biosynthesis and/or activity of CYPs, maybe promoting the formation of such carcinogens and increasing their toxicity [115,116,117]. While CYPs are affected by flavonoids, their chemical structure can be affected by these enzymes as well. CYP-generated metabolites of flavonoids may bind to DNA and induce similar effects like mutagenic DNA alkylating agents [114]. Walle et al. 2003 documented the covalent binding of ROS-activated quercetin to DNA and proteins in several cancer cell lines [118]. This binding to the DNA induces the destabilization of the helix [119] and may result in apoptosis, cell cycle arrests or mutations. The carcinogenic potential of quercetin has already shown in rats by the formation of kidney tumors, where the authors assumed the combination of genotoxic and non-genotoxic effects [120]. In addition to the DNA alkylating potential, clastogenic activity was reported for several flavonoids, and they seemed to appear independently from each other [121]. Similar to flavonoids, a wide range of polyphenols are able to induce toxic effects, e.g., by an increase of the mutagenicity of *N*-Nitrosopyrrolidine, a nitrosamine mainly occurring in nitrite-rich food after cooking [122]. In all these studies, the evaluation of the toxic potential of phenolic compounds was done in vitro using cancer cell lines or in vivo with rats. Therefore, the transfer to human beings is limited and need to be verified in further experiments [123]. Nevertheless, the above-mentioned toxic effects depend on the bioaccessibility and bioavailability of phenolic compounds and will be strengthen by an encapsulation. Finally, the benefits as well as the risks of phenolic compounds should be reconsidered prior to application.

## 8. Conclusions

In this review, the effects of encapsulation techniques on the bioaccessibility and bioavailability of phenolic compounds were discussed. Emulsion/nanoemulsion, solid lipid nanoparticles and liposomes are the most important lipid-based delivery systems that are used to increase the retention of phenolic compounds during gastrointestinal digestion and cellular uptake. Overall, findings in the literature suggest that encapsulation is a promising tool that could be used for higher stability and greater retention of phenolic compounds.

As a future aspect, it could be suggested that more sophisticated in vitro as well as in vivo tests should be conducted to stimulate the physicochemical change and digestion process of the formulation in the entire digestive tract. The bioaccessibility and bioavailability of many dietary phenolic compounds are not well defined. The potential biological activity of each compound and their metabolites should be investigated and compared to get a better approach to assess the effects of encapsulated bioactives on the human digestive system.

## Figures and Tables

**Table 1:** Effects of different lipid-based encapsulation techniques on the bioaccessibility of selected phenolic compounds.

Applied Technique	Active Material	Carrier	Results	References
Nanoemulsion/ emulsion	Curcumin	Triacylglycerol	1 to 58% bioaccessibility of nanoemulsion-based delivery systems	[20]
W/O/W emulsion gels	EGCG and Quercetin	Gelatin	After coencapsulation in W/O/W emulsion gels, 48.4 and 49% bioaccessibility of EGCG and quercetin, respectively	[57]
Nanostructured lipid carriers, Lipid nanoemulsions, Solid lipid nanoparticles	Quercetin	Lecithin	~60% bioaccessibility with nanostructured lipid carriers and lipid nanoemulsions, ~35% with solid lipid nanoparticles and ~7% with free quercetin solution	[33]
Nanoemulsion	Quercetin	Triacylglycerol	An enhancement in the quercetin bioaccessibility from <5% in bulk water to 53% in nanoemulsions	[38]
Nanostructured lipid carrier	Quercetin	Glyceryl monostearate, glycerol monolaurate and caprylic capric triglyceride	33.6 and 2% bioaccessibility of quercetin in nanostructured lipid carrier and bulk water, respectively	[58]
Solvent displacement method	Quercetin	Eudragit	7 and 22% release of quercetin in water and polymeric nanoparticles, respectively	[59]
Nanoemulsion	Resveratrol	Peanut oil	No changes in the quantity and quality of the resveratrol-loaded nanoemulsions	[37]
Antisolvent precipitation/emulsion	Tangeretin	Zein and β-lactoglobulin	15 to 37% bioaccessibility of tangeretin without and 4% initial oil concentration, respectively	[60]
Viscoelastic emulsion	Tangeretin	MCT	According to in vitro lipolysis, 9.7 to 29.3% release of tangeretin within oil suspension and emulsion, respectively. According to TIM-1 model, 2.6-fold increase in tangeretin bioaccessibility within emulsion system	[61]
High internal phase emulsions	Tangeretin	Whey protein isolate—low methoxy pectin	According to in vitro lipolysis, 2-fold increase in bioaccessibility within HIPE-complexes compared to that of the bulk oil According to TIM-1 model, 5-fold increase in bioaccessibility within HIPE-complexes compared to that of the bulk oil	[62]
Pickering emulsion	5-DN	Peanut protein	9.2 and 18.3% release of 5-DN in bulk oil and emulsion, respectively	[63]
High internal phase emulsions	Nobiletin	Whey protein isolate—low methoxy pectin	According to in vitro lipolysis, 1.5-fold increase in bioaccessibility within HIPE-complexes compared to that of the bulk oil According to TIM-1 model, 2-fold increase in bioaccessibility within HIPE-complexes compared to that of the bulk oil	[62]
Nanoemulsion/ Pickering emulsion	PMFs extract	MCT	According to in vitro lipolysis, 14-fold increase in bioaccessibility within nanoemulsion/emulsion compared to that of the bulk oil According to TIM-1 model, 2- and 4-fold increase in bioaccessibility within nanoemulsion and emulsion, respectively, compared to that of the bulk oil	[64]

TIM-1: in vitro dynamic digestion model; HIPE: high internal phase emulsions; 5-DN: 5-demethylnobiletin; EGCG: (−)-epigallocatechin-3-gallate, PMF: polymethoxylated flavonoids; W/O/W: water-in-oil-in-water emulsion.

**Table 2:** Effects of lipid-based encapsulation techniques on the in vitro bioavailability of phenolic compounds.

Applied Technique	Active Material	Carrier	In Vitro Model: Analyzed Material and *Results*	References
Antisolvent precipitation	Quercetin	Shellac and almond gum	Caco-2 absorption study: analysis of intracellular quercetin level *Results of the cellular uptake could not be compared with nonencapsulated quercetin sample due to cytotoxic effects of nanoparticles*	[102]
Emulsion−diffusion solvent evaporation	Quercetin	Poly(lactic-co-glycolic acid)	Caco-2 absorption study: analysis of intracellular quercetin level *6-fold higher uptake efficiency by encapsulation*	[95]
Self-nanoemulsion	Quercetin	Castor oil	Caco-2 monolayer transport system: analysis of the supernatant from apical and basolateral compartment *Encapsulation enabled a 2-fold higher transportation of quercetin*	[103]
Pickering emulsion	Curcumin	Milled starch particles	Caco-2 absorption study: analysis of intracellular curcumin level *Encapsulation enabled a significantly higher uptake efficiency*	[64]
Antisolvent precipitation/emulsion	Tangeretin	Zein and β-lactoglobulin	Caco-2 monolayer transport system: analysis of the supernatant from apical and basolateral compartment *Oil dose-dependent increase in permeability*	[60]
Pickering emulsion	5-DN	Peanut protein	Caco-2 absorption study: analysis of intracellular 5-DN level by HPLC *Higher uptake rate of 5-DN by encapsulation* Caco-2 monolayer transport system: analysis of the supernatant from apical and basolateral compartment *Higher permeability of 5-DN micelles than nonencapsulated 5-DN*	[63]
O/W nanoemulsion	Resveratrol	Lipophilic soy lecithin with defatted soy lecithin and peanut oil	Caco-2 monolayer transport system: analysis of the supernatant from apical and basolateral compartment *Significantly lower permeability of encapsulated resveratrol*	[104]
O/W nanoemulsion	Resveratrol	Soy lecithin with peanut oil	Caco-2 absorption study: analysis of intracellular resveratrol level *Significantly higher uptake of resveratrol by encapsulation* Caco-2 monolayer transport system: analysis of the supernatant from apical and basolateral compartment *Significantly lower permeability of encapsulated resveratrol*	[104]
O/W nanoemulsion	Resveratrol	Lipophilic soy lecithin with peanut oil	Caco-2 absorption study: analysis of intracellular resveratrol level *Significantly lower uptake of resveratrol by encapsulation*	[104]
O/W nanoemulsion	Resveratrol	Tween 20 with glycerol monooleate and peanut oil	Caco-2 absorption study: analysis of intracellular resveratrol level *Significantly lower uptake of resveratrol by encapsulation* Caco-2 monolayer transport system: analysis of the supernatant from apical and basolateral compartment *Significantly lower permeability of encapsulated resveratrol*	[104]

**Table 3:** Effects of lipid-based encapsulation techniques on the in vivo bioavailability of phenolic compounds.

Applied Technique	Active Material	Carrier	In Vivo Model: Analyzed Material and *Results*	References
Emulsification/thermal gelation	Anthocyanins from bilberry extract	Whey protein	Humans: analysis of serum and urine samples *28% less anthocyanins in serum but 108% more anthocyanins in urine than nonencapsulated extract*	[92]
Emulsification/thermal gelation	Anthocyanins from bilberry extract	Citrus pectin	Humans: analysis of serum and urine samples *80% less anthocyanins in serum and 8% less anthocyanins in urine than nonencapsulated extract*	[92]
Ionic gelation	Chlorogenic acid	Chitosan nanoparticles	Rats: analysis of serum samples *Encapsulation enabled a slower and sustained absorption*	[107]
Spray drying	Polyphenol extract from cocoa nibs	High-amylose maize starch	Humans: analysis of serum and urine samples *Significantly lower content of phenolic acids in serum and urine induced by encapsulation of polyphenols*	[91]
O/W emulsion	Resveratrol	Sodium caseinate with high amylose maize starch and glucose	Rats: analysis of radiolabelled [^3^H]-resveratrol along digestive system *Encapsulation led to a significantly higher content of resveratrol in the gut lumen of small intestine*	[105]
Emulsion−diffusion solvent evaporation	Quercetin	Poly(lactic-co-glycolic acid)	Rats: analysis of serum samples *2.9-fold higher uptake efficiency by encapsulation*	[95]
Self-nanoemulsion	Quercetin	Castor oil	Rats: analysis of serum samples *2-fold significantly higher uptake efficiency by encapsulation*	[103]
Organogel-based nanoemulsion	Curcumin	Organogel	Mice: analysis of serum samples *Encapsulation led to a 9-fold higher bioavailability of curcumin*	[110]
Sophorolipid-coated nanoparticle	Curcumin	Sophorolipid micelles	Rats: analysis of serum samples *Significantly higher (3.6-fold) absorption of encapsulated curcumin*	[90]
Bowman−Birk inhibitor nanodeliverycarrier	Curcumin	Soybean	Rats: analysis of serum samples *Encapsulation with Bowman−Birk inhibitor led to a slower but significantly 3.1-fold higher uptake compared to curcumin-loaded sodium caseinate nanoparticles*	[109]
Fluidized bed spray coating	Curcumin	Cellulose derivative with vegetable oil	Humans: analysis of serum samples *7.3-fold higher content of curcuminoids in serum, while the urinary concentration was not significantly affected by encapsulation*	[108]
Casein nanoparticle	Resveratrol	Sodium casein	Rats: analysis of serum samples *10-fold higher bioavailability of encapsulated resveratrol than dissolved in polyethylenglykol*	[106]
